# Supplementary material for: KMD clustering: robust general-purpose clustering of biological data
Source: Commun Biol. 2023 Nov 2;6:1110. doi: 10.1038/s42003-023-05480-z (PMC10622433; doi:10.1038/s42003-023-05480-z)
Supplement: Supplementary file 2 — Supplemental information [file 42003_2023_5480_MOESM2_ESM.pdf]

Supplementary information

Supplementary tables

	nested circles<br>(1000 objects, 2 clusters)			half moons<br>(1000 objects, 2 clusters)			globular clusters<br>(1000 objects, 3 clusters)			anisotropic clusters<br>(1000 objects, 3 clusters)		
	Accuracy	NMI	ARI	Accuracy	NMI	ARI	Accurac y	NMI	ARI	Accurac y	NMI	ARI
Spectral	1.	1.	1.	1.	1.	1.	0.983	0.925	0.95	0.949	0.838	0.853
Average Linkage	0.661	0.224	0.103	0.897	0.612	0.63	0.983	0.921	0.95	0.883	0.694	0.699
Single Linkage	1.	1.	1.	1.	1.	1.	0.335	0.004	0.	0.335	0.004	0.
DBSCAN	0.821	0.72	0.733	1.	1.	1.	0.97	0.911	0.941	0.985	0.968	0.977
HDBSCAN	1.	1.	1.	0.999	0.99	0.996	0.984	0.935	0.96	1.	1.	1.
Gaussian Mixture	0.508	0.	-0.001	0.852	0.396	0.495	0.982	0.917	0.947	0.999	0.994	0.997
KMD	1.	1.	1.	1.	1.	1.	0.979	0.915	0.938	0.991	0.955	0.973
KMD core	1.	1.	1.	1.	1.	1.	0.983	0.924	0.952	1.	1.	1.
KMD chosen K	5			99			36			56		

Table S1. Evaluation on simulated datasets. Evaluation of algorithms by accuracy, Normalized Mutual Information, Adjusted Rand Index. Comparison of clustering algorithm performance on standard scikit-learn simulated datasets. Algorithms: Spectral clustering, hierarchical clustering average linkage, hierarchical clustering single linkage, DBSCAN, gaussian mixture, and KMD clustering and KMD clustering core clusters. Datasets: nested circles, half-moons, globular clusters and anisotropic clusters.

	nested circles (1000 objects, 2 clusters)			half moons (1000 objects, 2 clusters)			globular clusters (1000 objects, 3 clusters)			anisotropic clusters (1000 objects, 3 clusters)		
	Accuracy	NMI	ARI	Accuracy	NMI	ARI	Accuracy	NMI	ARI	Accuracy	NMI	ARI
Spectral	0.711	0.289	0.177	0.853	0.403	0.5	0.899	0.665	0.726	0.91	0.741	0.76
Average Linkage	0.687	0.237	0.139	0.839	0.41	0.459	0.632	0.514	0.428	0.668	0.722	0.568
Single Linkage	0.501	0.002	0	0.501	0.002	0	0.335	0.004	0	0.335	0.004	0
DBSCAN	0.667	0.536	0.721	0.494	0.352	0	0.342	0.033	0	0.67	0.724	0.567
HDBSCAN	0	0	0	0.941	0.678	0.778	0.924	0.754	0.794	0.667	0.736	0.569
Gaussian Mixture	0.552	0.009	0.011	0.834	0.353	0.457	0.923	0.727	0.784	0.998	0.976	0.988
KMD	0.990	0.922	0.960	0.915	0.581	0.689	0.914	0.717	0.763	0.971	0.881	0.916
KMD core	0.992	0.932	0.967	0.929	0.631	0.736	0.916	0.726	0.772	0.995	0.973	0.984
KMD chosen K	17			90			27			99		

Table S2. Evaluation on simulated noisy datasets. Evaluation of algorithms by accuracy, Normalized Mutual Information, Adjusted Rand Index. Comparison of clustering algorithm performance on standard scikit-learn simulated datasets with high noise added. Algorithms: Spectral clustering, hierarchical clustering average linkage, hierarchical clustering single linkage, DBSCAN, gaussian mixture,and KMD clustering and KMD clustering core clusters. Datasets: nested circles, half-moons, globular clusters and anisotropic clusters.

	**nested circles** (1000 objects, 2 clusters)			**half moons** (1000 objects, 2 clusters)			**globular clusters** (1000 objects, 3 clusters)			**anisotropic clusters** (1000 objects, 3 clusters**)**		
	Accuracy	NMI	ARI	Accuracy	NMI	ARI	Accuracy	NMI	ARI	Accuracy	NMI	ARI
Average Linkage	0.687	0.237	0.139	0.839	0.41	0.459	0.632	0.514	0.428	0.668	0.722	0.568
Single Linkage	0.501	0.002	0	0.501	0.002	0	0.335	0.004	0	0.335	0.004	0
Complete Linkage	0.804	0.364	0.369	0.834	0.367	0.446	0.863	0.621	0.643	0.582	0.471	0.376
Ward Linkage	0.662	0.184	0.104	0.756	0.205	0.261	0.903	0.694	0.729	0.844	0.741	0.652
Minimax Linkage	0.699	0.253	0.158	0.742	0.205	0.234	0.890	0.660	0.698	0.680	0.598	0.475
KMD	0.990	0.922	0.960	0.915	0.581	0.689	0.914	0.717	0.763	0.971	0.881	0.916
KMD core	0.992	0.932	0.967	0.929	0.631	0.736	0.916	0.726	0.772	0.995	0.973	0.984

Table S3. Evaluation of different linkage methods on simulated noisy datasets.

Evaluation of linkage methods by accuracy, Normalized Mutual Information, Adjusted Rand Index. Comparison of clustering algorithm performance on standard scikit-learn simulated datasets with high noise added.

	**Levine15_13**<br><br>(9887 out of 167,044 Cells, 13 markers, 24 clusters)			**Levine15_32**<br><br>(7881 out of 265,627 Cells, 32 markers, 14 clusters)			**Samusik16**<br><br>(12199 out of 86,864 Cells, 39 m arkers,24 clusters)		
	accuracy	NMI	ARI	accuracy	NMI	ARI	accuracy	NMI	ARI
kmeans	0.5587+-0.0303	0.7071+-0.0139	0.5642+-0.0367	0.5702+-0.0550	0.7252+-0.0213	0.6169+-0.0755	0.4819+-0.0585	0.6506+-0.0201	0.4628+-0.0512
Xshift	0.7824+-0.0216	0.7849+-0.0205	0.7722+-0.0298	0.8706+-0.0510	0.8646+-0.0405	0.8958+-0.04278	0.9100+-0.0230	0.8702+-0.0063	0.8890+-0.0281
DEPECHE	0.6918+-0.0141	0.6679+-0.0094	0.6559+-0.0107	0.8922+-0.0023	0.8416+-0.0030	0.9273+-0.0031	0.8253+-0.0067	0.7243+-0.0059	0.8251+-0.0079
Accense	0.5591 +-0.1203	0.7224 +-0.061	0.5911 +-0.129	0.5218+-0.0947	0.6644+-0.0590	0.4811+-0.1048	0.5935+-0.0714	0.7279+-0.0383	0.5664+-0.0767
FlowSOM	0.8436+-0.0272	0.8443+-0.0143	0.8579+-0.0209	0.8646+-0.0903	0.8725+-0.0596	0.8734+-0.1195	0.6331+-0.0666	0.6599+-0.0460	0.6188+-0.0833
phenoGraph	0.9180+-0.0014	0.8827+-0.0021	0.9268+-0.0032	0.6598+-0.0412	0.7607+-0.0220	0.6719+-0.0368	0.9235+-0.0423	0.8996+-0.0257	0.9248+-0.0524
KMD	0.8384+-0.0092	0.8507+-0.0177	0.7963+-0.0208	0.9296+-0.0105	0.9357+-0.0066	0.9594+-0.0070	0.8940+-0.0037	0.8412+-0.0050	0.8626+-0.0063
KMD chosen k	K={96,99,89,91,99}			K={11,96,96,96,11}			K={96,96,96,96,96}		

Table S4. Evaluation on mass cytometry data. Average performance of six clustering algorithms: kmeans, Xshift, DEPECHE, Accense, FlowSOM, Phenograph, flowMeans and KMD clustering by accuracy, Normalized Mutual Information Adjusted Rand Index with standard deviation.

	**nested circles** (1000 objects, 2 clusters)			**half moons** (1000 objects, 2 clusters)			**globular clusters** (1000 objects, 3 clusters)			**anisotropic clusters** (1000 objects, 3 clusters**)**		
	Accuracy	NMI	ARI	Accuracy	NMI	ARI	Accuracy	NMI	ARI	Accuracy	NMI	ARI
Louvain	0.19	0.387	0.142	0.187	0.305	0.131	0.0347	0.594	0.313	0.296	0.48	0.247
Leiden	0.138	0.350	0.100	0.155	0.268	0.089	0.232	0.535	0.218	0.213	0.442	0.176
Average SCCAF	0.56	0.348	0.292	0.553	0.152	0.068	0.675	0.642	0.522	0.460	0.206	0.150
KMD	0.990	0.922	0.960	0.915	0.581	0.689	0.914	0.717	0.763	0.971	0.881	0.916
KMD core	0.992	0.932	0.967	0.929	0.631	0.736	0.916	0.726	0.772	0.995	0.973	0.984

Table S5. Evaluation of different single-cell clustering methods on simulated datasets. Evaluation of methods by accuracy, Normalized Mutual Information, Adjusted Rand Index. Comparison of clustering algorithm performance on standard scikit-learn simulated datasets with high noise added. SCCAF was run 10 times to offset the effect of randomness.

	Simulated scRNA-seq (150 objects, 3 clusters)		
	Accuracy	NMI	ARI
Louvain	0.993	0.970	0.989
Leiden	0.987	0.950	0.960
KMD	0.987	0.940	0.960

Table S6. Evaluation of different single-cell clustering methods on simulated scRNA-seq dataset. Evaluation of methods by accuracy, Normalized Mutual Information, Adjusted Rand Index. Comparison of clustering algorithm performance on a simulated scRNA-seq dataset by Townes, et al. (doi:10.1186/s13059-019-1861-6).

	**Lawlor17** (638 cells,19927 genes,8 clusters)			**Zeisel15** (2361 cells, 2000 genes, 7 clusters)			**Li17** (561 cells, 25083 genes, 7 clusters)		
	accuracy	NMI	ARI	accuracy	NMI	ARI	accuracy	NMI	ARI
Louvain	0.674	0.699	0.576	0.623	0.728	0.496	0.729	0.764	0.592
Leiden	0.686	0.723	0.583	0.52	0.683	0.400	0.713	0.783	0.586
Average SCAAF	0.808	0.76	0.769	0.711	0.731	0.586	0.582	0.5	0.341
Seurat	0.657	0.678	0.658	0.611	0.716	0.488	0.715	0.687	0.544
KMD	0.893	0.790	0.831	0.738	0.686	0.523	0.925	0.882	0.885
KMD chosen K	K=15			K=4			K=2		

Table S7. Evaluation on scRNA-seq datasets. performance of four clustering algorithms: Louvain, Leiden, SCCAF, Seurat and KMD. Metrics chosen are: clustering accuracy, Normalized Mutual Information, Adjusted Rand Index.

Supplementary figures

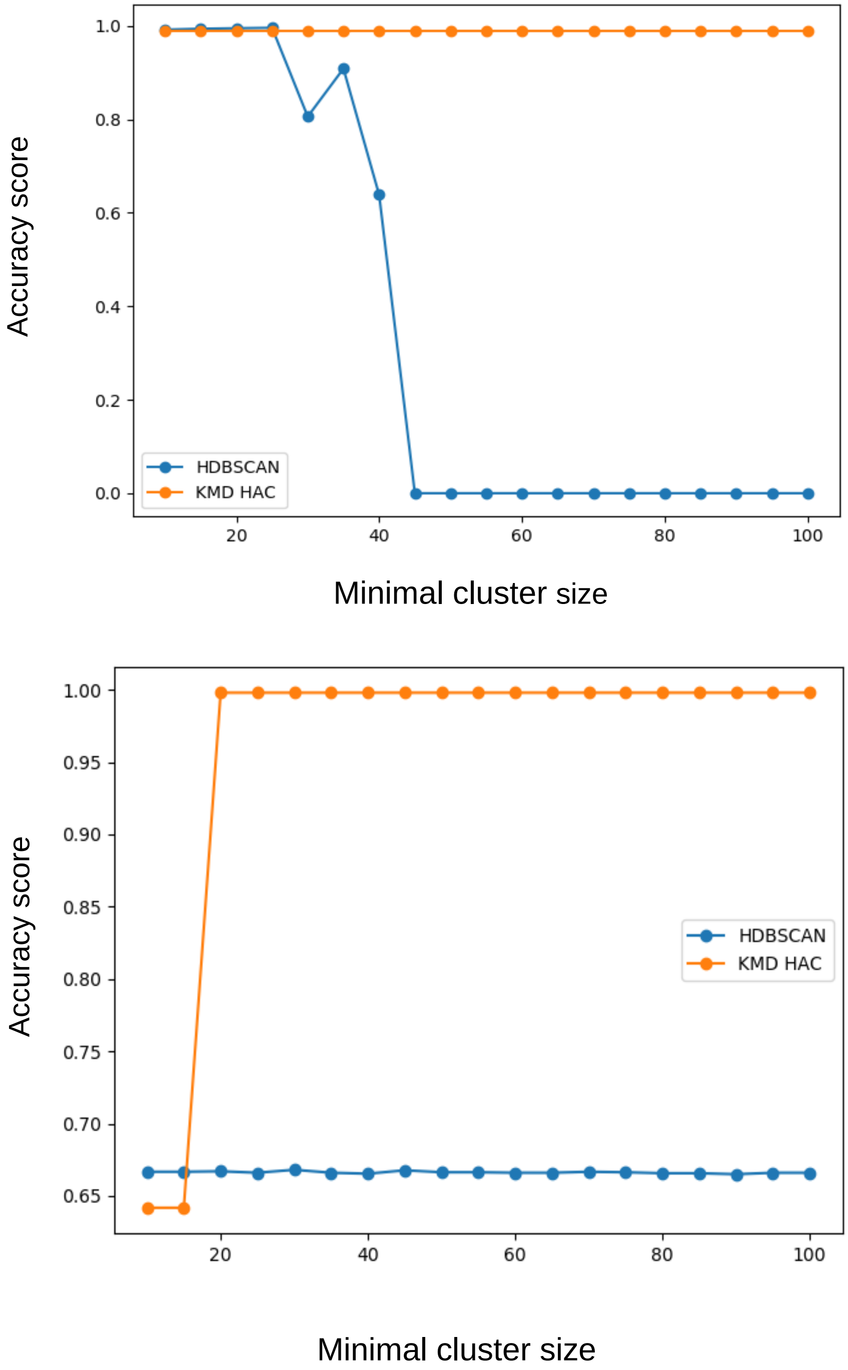

Figure S1. Effect of minimal cluster size of clustering accuracy. Clustering accuracy of KMD clustering and HDBSCAN on the high-noise nested circles (top) and high noise anisotropic clusters (bottom) datasets, shown for a range of minimal cluster size values.

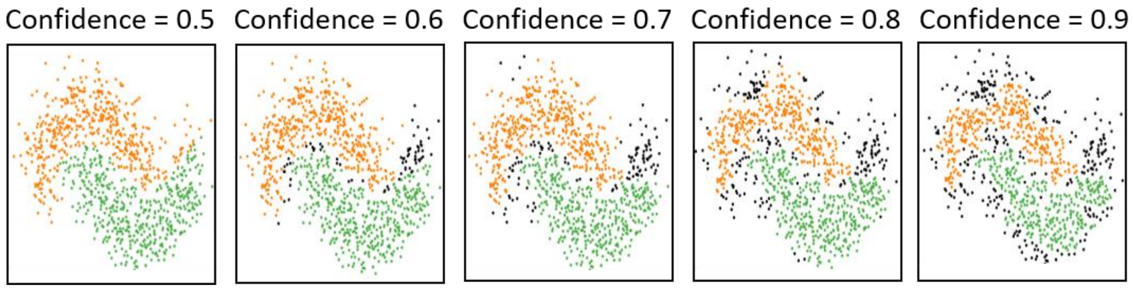

Figure S2. Effect of outlier assignment confidence threshold. Outlier cluster assignment at various confidence thresholds.

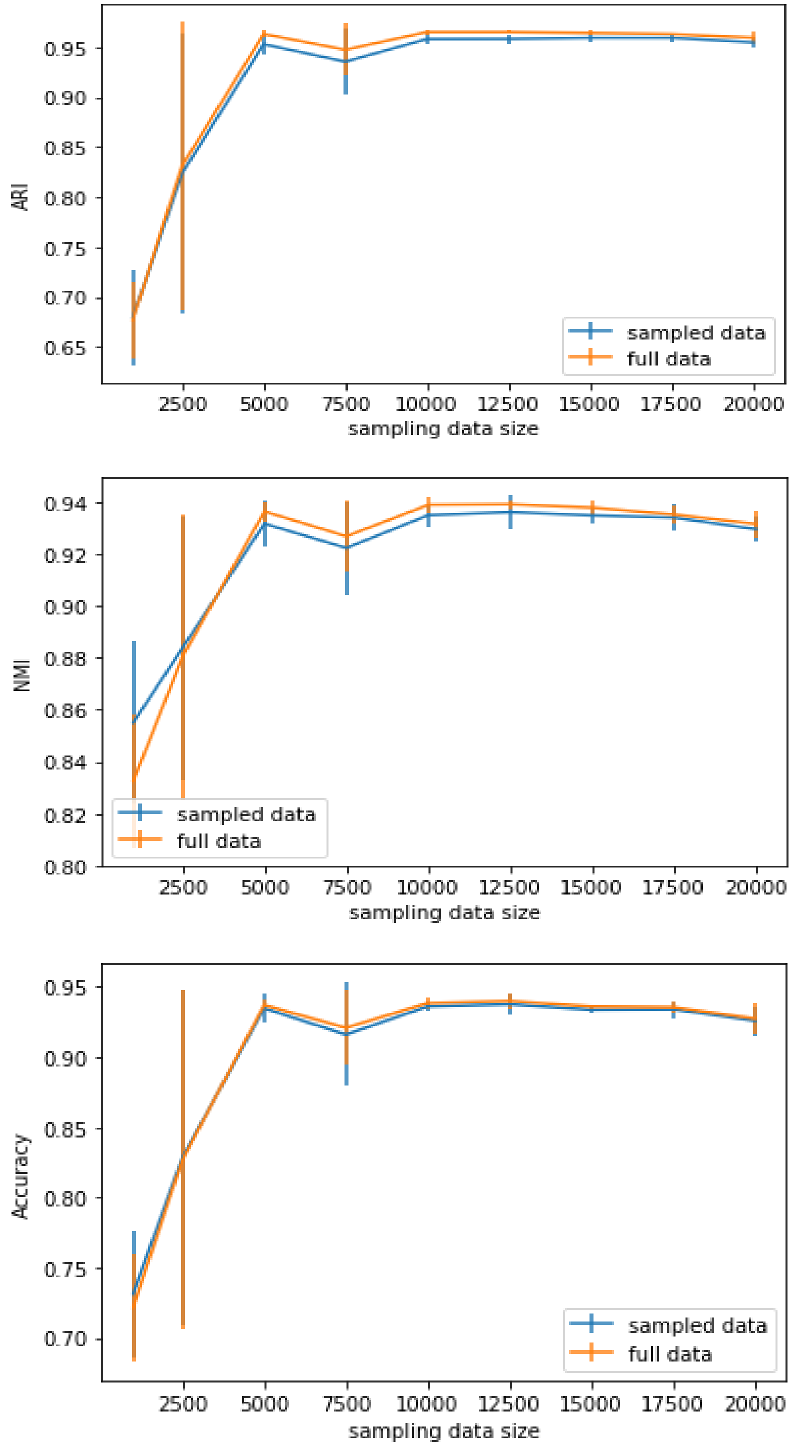

Figure S3. Clustering performance using sampling-based approach. Sampling-based KMD clustering was performed on the full Levine32dim dataset by randomly sampling a subset of cells of the specified sizes (1000-20000). ARI, NMI and accuracy were evaluated both for the sampled subset and for the entire data (i.e. the sampled subset which for the core clusters as well as the rest of the cells which were assigned based on the core clusters).

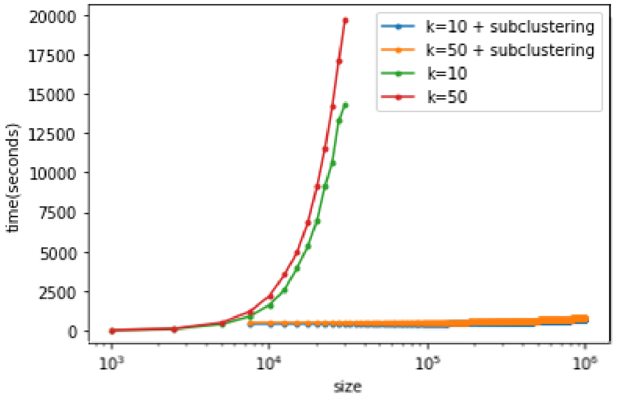

Figure S4. KMD clustering run time on large dataset. KMD clustering runtime was evaluated on random samples taken from the simZeisel15 dataset which contains 1M objects. Two different k values (10 and 50) are shown. Full KMD clustering was performed for datasets of up to 20000 objects. Sampling-based KMD clustering (marked “subclustering”) was performed on datasets of up to 1M objects, while the sample size was kept constant at 5000 objects.
